# Integrative pan-cancer analysis reveals the prognostic and immunotherapeutic value of ALKBH7 in HNSC

**DOI:** 10.18632/aging.205981

**Published:** 2024-06-29

**Authors:** Tianyu Wang, Bojian Lin, Boyu Cai, Zhiwen Cao, Caiquan Liang, Shunyu Wu, Enhong Xu, Li Li, Hu Peng, Huanhai Liu

**Affiliations:** 1 Department of Otolaryngology-Head and Neck Surgery, The Second Affiliated Hospital of Naval Medical University, Naval Medical University, Shanghai 200003, P.R. China; 2 Department of Health Management Center, The First Affiliated Hospital of Naval Medical University, Shanghai 200438, P.R. China; 3 Department of Otolaryngology, Jinshan Hospital, Fudan University, Shanghai 201508, P.R. China; 4 Department of Otolaryngology-Head and Neck Surgery, Ninth People’s Hospital, Shanghai Jiao Tong University School of Medicine, Shanghai 200011, P.R. China; 5 Department of Otolaryngology, Naval Medical Center, Naval Medical University, Shanghai 200052, P.R. China

**Keywords:** pan-cancer, ALKBH7, head and neck squamous cell carcinoma, tumor cell proliferation, biomarker, prognosis

## Abstract

The AlkB homolog 7 (ALKBH7) is a nonheme iron (II) α-ketoglutarate-dependent dioxygenase superfamily member, which may affect the progression of several types of human cancer. However, the biological effect, especially the immune-related effect, of ALKBH7 in HNSC remains unclear. Herein, several databases were employed at first to assess the different expression of ALKBH7 as well as their relationship to the prognosis, RNA modification, DNA methylation modulation, immune microenvironment and chemotherapeutic responses of various types of cancers. We found that ALKBH7 was expressed differentially in pan-cancer, and correlated with a satisfied prognosis especially in HNSC. The expression of ALKBH7 was also associated with the level of immune cell infiltration, TMB, MSI, HRD, MMR deficiency, and DNA methyltransferases in a wide variety of cancers, which might be potentially related to the responses against chemotherapeutic agents. Next, the role of ALKBH7 in HNSC was further investigated. Western blot and immunohistochemical analysis in HNSC patients from the NMU cohort showed the reduced ALKBH7 expression level in tumor tissues. *In vitro* experiments of cell migration, invasion, and proliferation showed a potential protective effect of ALKBH7 in HNSC. Collectively, ALKBH7 might play a protective role in the development and progression of multiple cancers by affecting the metabolism and immune cell infiltration, especially HNSC, which could be a potential biomarker to predict prognosis and chemotherapeutic response.

## INTRODUCTION

Head and neck squamous cell carcinoma (HNSC) is the sixth most common cancer globally, derived from the mucosal epithelium in the oral cavity, pharynx, larynx, and sinonasal tract [1, 2]. The incidence of HNSC continues to rise and is expected to increase 1.08 million new cases annually by 2030 [3, 4]. Epidemiological studies have revealed the risk factors of HNSC include tobacco consumption, alcohol consumption, viral infection and exposure to environmental pollutants, and The Cancer Genome Atlas has revealed that HNSC is characterized by genetic instability and epigenetic changes in a multistep process [5]. Although the treatment of HNSC has been improved by immunotherapies as well as epidermal growth factor receptor (EGFR)-based targeted therapies, the prognosis of HNSC patients still remains poor, with about 50% of patients suffered from acquired therapies resistances or tumor metastasis due to unclear mechanisms [6–9]. Thus, further investigation against the HNSC development is required to predict the progression of premalignant HNSC lesions, prognosticate survival, revealing new intervention targets, and predicting response to therapeutic agents.

The AlkB homolog 7 (ALKBH7), a member of the nine human homologs of the Alkane monooxygenase (AlkB) family, is a nonheme Fe (II) and α-ketoglutarate (α-KG) dependent dioxygenase, which is located in mitochondria [10–13]. ALKBH7 is required for alkylation- and oxidation-induced programmed necrosis and has been reported to act as a key player in the process of the DNA damage response, mitochondrial membrane potential homeostasis, mitochondrial dysfunction and fatty acid metabolism [12–14]. It has been well documented that ALKBH7 could down regulate the expression of its key effectors like UQCRH and HMGN1 and thereby modulate the cell proliferation, lipid metabolism and programmed necrosis in a variety of solid tumors including prostate cancer, ovarian serous carcinoma, hepatocellular carcinoma, breast carcinoma, non-small cell lung cancer and glioblastoma, indicating ALKBH7 may be a potential prognostic maker in a series of solid cancers [15–23]. However, how the ALKBH7 regulate the cancer progression and whether it is related to the prognosis of head and neck cancer has not been well understood yet. Therefore, a deep investigation of ALKBH7 in cancers is still required to verify whether it is an appropriate biomarker in prognosis of cancers.

In this study, we analyzed the role of ALKBH7 in pan-cancer by using public multi-databases. The analysis against gene expression, its DNA methylation as well as RNA modification of ALKBH7 and its relationship to the prognosis and the tumor microenvironment such as TMB, MSI, HRD, and MMR of cancers was performed at first. Next, the role of ALKBH7 in immunoregulation and tumor microenvironment development was evaluated and the correlation between ALKBH7 and therapy resistance was also monitored. Furthermore, the function of ALKBH7 to inhibit the growth and progression of HNSC cells was determined *in vitro*. Our result showed increased expression of ALKBH7 may have a protective effect on the development and progression of various cancers, particularly HNSC, by influencing metabolism and immune cell infiltration.

## RESULTS

### The expression of ALKBH7 was significantly different and associated with clinical outcomes in multiple cancers, especially in the HNSC

**Figure 1:**
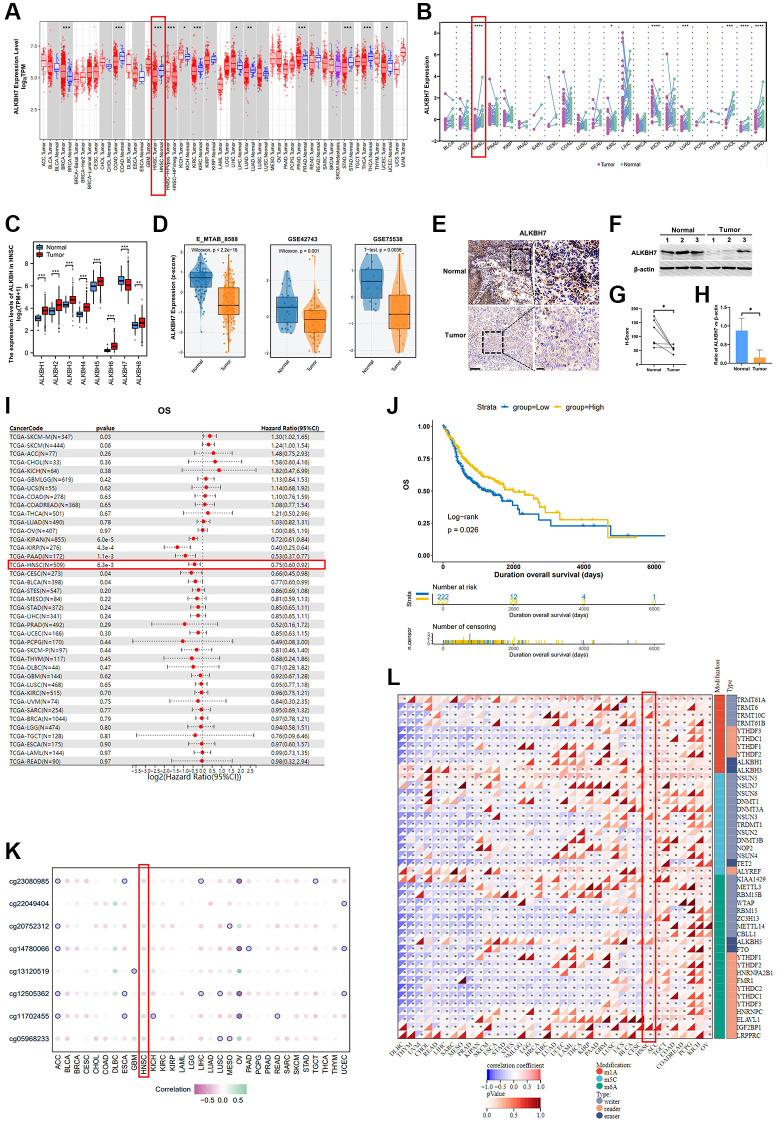
**The clinical correlation of ALKBH7 expression and the modification related to ALKBH7 expression in pan cancer, especially in HNSC**. (**A**) Differential expression of ALKBH7 in 33 cancer types and (**B**) in paired tumor and normal tissues of 22 cancer types from the TCGA dataset. (**C**) The expression levels of ALKBH in tumor tissue and adjacent normal tissue from HNSC patients. (**D**) Differential expression of ALKBH7 in HNSC from various datasets. (**E**) Representative immunohistochemical images of ALKBH7 expression in HNSC and adjacent tissues from Second Affiliated Hospital of Naval Medical University, scale bar = 100 μm (left); 20 μm (right). (**F**) Western blotting of ALKBH7 differential expression in tumor tissue and adjacent normal tissue from HNSC patients. (**G**) Quantification of the H-score for ALKBH7 protein expressed level assessed by immunohistochemical assay. (**H**) Quantitative ratio of gray value for ALKBH7 protein expressed level assessed by western blotting. (**I**) Univariate Cox regression of ALKBH7 for OS in pan-cancers. (**J**) Correlation between ALKBH7 expression and OS in HNSC patients from TCGA dataset. (**K**) The correlation between ALKBH7 expression and DNA methylation degree in pan cancer. (**L**) The correlation between ALKBH7 expression and RNA modification regulator expression in pan cancer. ^*^p < 0.05, ^**^p < 0.01, ^***^p < 0.001, ^****^p < 0.0001.

Applying TCGA database for pan-cancer analysis, we found that in COAD, HNSC, KIRC, LUAD, STAD, THCA, and UCEC, ALKBH7 expression in tumor tissues was lower than that in normal tissues. In contrast, ALKBH7 was highly expressed in BRCA, KICH, LIHC, and PRAD tumor tissues (Figure 1A, 1B). The further analysis was conducted to supplementally show the expressions of ALKBH7 in different cancer cells and normal tissues according to the CCLE and the GTEx datasets (Supplementary Figure 1A, 1B). To focus on HNSC, the differential expression of the ALKBH family between tumor and normal tissues was determined based on the TCGA dataset and outhouse datasets of HNSC. We found that only level ALKBH7 reduced in tumor tissues (Tumor) than that in the adjacent tissues (Normal), although the other ALKBH family genes of elevated during the tumor development (Figure 1C), while the outhouse datasets of HNSC (E_MTAB _8588, GSE42743, and GSE75538) also illustrated the reduction of ALKBH7 expression in HNSC tumor tissues (Figure 1D), indicating that ALKBH7 might be involved in HNSC tumor growth and development. The GEO datasets (GSE42743) also exhibited significance of ALKBH7 level in different stages of HNSC, suggesting that ALKBH7 might play an essential role in tumorigenesis (Supplementary Figure 1C). Additionally, as HPV infection is recognized as an independent risk factor for laryngeal cancer, we analyzed additional datasets (GSE117973) based on HPV infection status, finding that ALKBH7 expression was increased in HPV positive cancer tissues (Supplementary Figure 1D). The additional clinical characteristics of high and low expression of ALKBH7 in HNSC patients based on TCGA were summarized in Supplementary Table 1. We further investigated the expression of ALKBH7 in different anatomic neoplasm subdivision of HNSC based on TCGA datasets, finding that the expression level of ALKBH7 was similar in various anatomical sites, but slightly higher in larynx than in mouth (Supplementary Figure 1E). Besides, the tissue samples from HNSC patients were also employed to validate the reduced level of ALKBH7 in HNSC tumors than that in adjacent tissues (Figure 1E–1H, Supplementary Figure 1F).

To further confirm whether the level of ALKBH7 was related to the better overall survival (OS) in HNSC, the univariate Cox regression of ALKBH7 for OS in pan-cancers was performed and the result showed that the highly expressed ALKBH7 indicated a satisfactory prognosis in KIPAN, KIRP, PAAD, HNSC, CESCN, and BLCA (Figure 1I). Afterwards, we determined the impact of the ALKBH7 level on overall survival (OS) in HNSC utilizing TCGA dataset, which also suggested positive correlation between ALKBH7 expression and better prognosis of HNSC patients (Figure 1J, Supplementary Figure 2A). Additionally, we investigated the effect of ALKBH7 expression on prognosis in different anatomic neoplasm subdivision of HNSC, finding that high expression of ALKBH7 was associated with a better prognosis in laryngeal squamous cell carcinoma compared to other types of HNSC (Supplementary Figure 2B–2F).

To further validate the protective biofunction of ALKBH7 in pan cancer, especially in HNSC, we systematically analyzed the potential regulation effect of DNA methylation and RNA modification in ALKBH7 expression. We found that DNA methylation could negatively affect ALKBH7 expression in multiple types of cancers (Figure 1K). For instance, the DNA methylation patterns of different loci could significantly decrease ALKBH7 expression in OV via cg23080985, cg14780066, cg12505362, and cg11702455. As for the correlation between RNA modifications (including m1A, m5C, and m6A) and ALKBH7 expression in pan-cancer (Figure 1L), we found that in ACC, TGCT, COAD, PCPG, KICH, and OV, RNA modification-related genes were generally positively correlated with the expression of ALKBH7, while they were negatively correlated in DLBC, THYM, UCM, CHOL, READ, LIHC and so on.

Altogether, the low expression level of ALKBH7 was observed in HNSC tissues compared to normal. The expression of ALKBH7 was negative correlated with the prognosis of patients with HNSC. DNA and RNA modification occurred at a pervasive level along with the reduction of ALKBH7 expression in multiple types of cancers. All these results suggested that ALKBH7 might be a protective gene in HNSC.

### Identifying ALKBH7 mainly impacted signaling pathways in pan-cancer, especially focusing on HNSC

**Figure 2:**
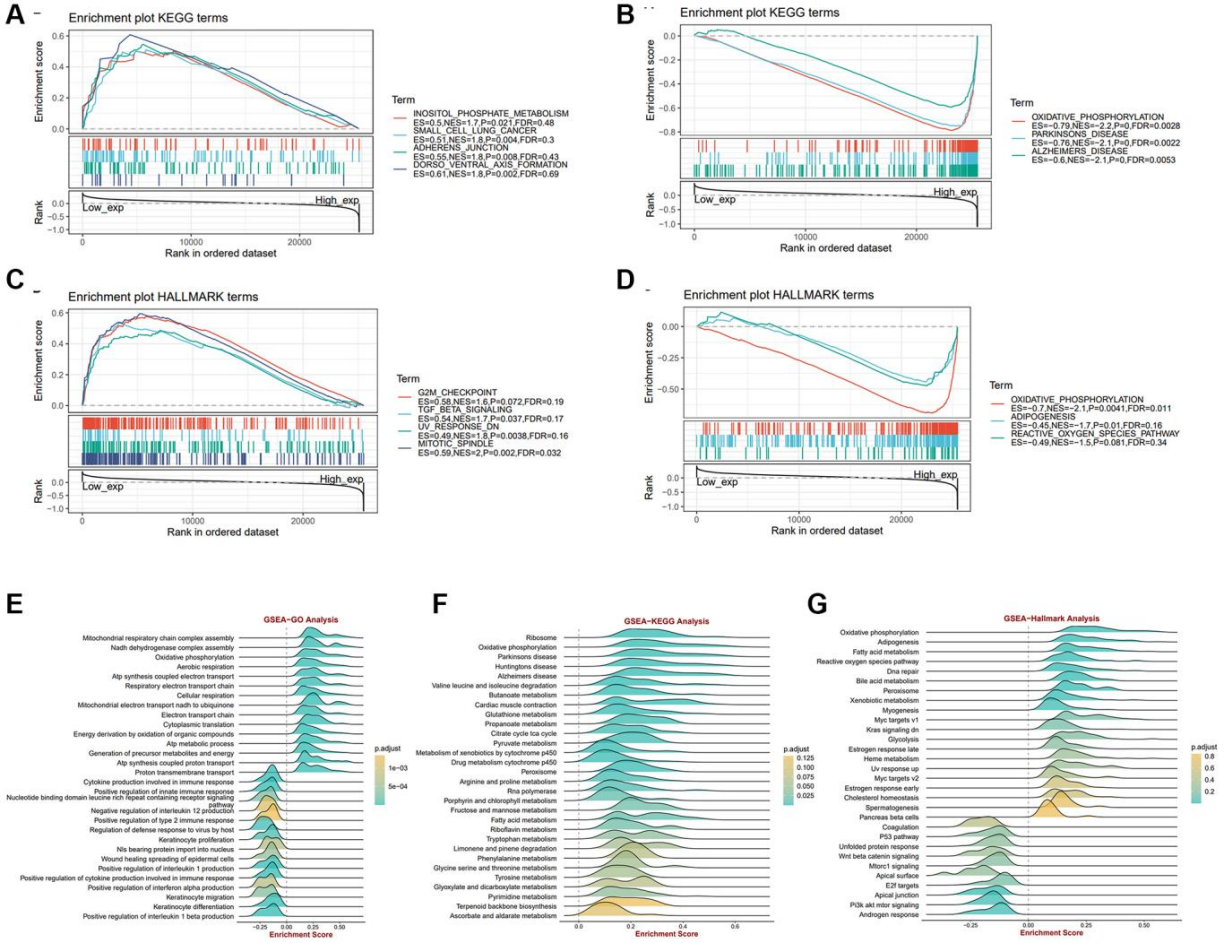
**The biological function analysis of ALKBH7 in pan-cancer from different datasets, especially in HNSC.** (**A**, **B**) Gene set enrichment analysis of ALKBH7 expression-related signaling pathways using the KEGG datasets in pan-cancer. (**C**, **D**) Gene set enrichment analysis of ALKBH7 expression-related signaling pathways using the HALLMARK datasets in pan-cancer. (**E**) Gene set enrichment analysis of ALKBH7-related biological functions using the GO datasets in HNSC. (**F**) Gene set enrichment analysis of ALKBH7-related signaling pathways using the KEGG datasets in HNSC. (**G**) Gene set enrichment analysis of ALKBH7-related signaling pathways using the HALLMARK datasets in HNSC.

To gain more insight into the biological process of ALKBH7 in cancers, gene set enrichment analysis (GSEA) and gene set variation analysis (GSVA) were performed based on the TCGA dataset (Supplementary Figure 3A, 3B). The findings revealed that ALKBH7 has a negative impact on oncogenic pathways, such as MTORC1 signaling, KRAS signaling, G2M checkpoint, and mitotic spindle pathways, while it promotes DNA repair pathways, suggesting a significant role of ALKBH7 in tumorigenesis. Moreover, ALKBH7 may influence tumor-associated metabolic pathways by upregulating oxidative phosphorylation and fatty acid metabolism pathways. The representative pathways identified by GSEA on KEGG and HALLMARK terms were shown in Figure 2A–2D. In the KEGG enrichment term, lowly expressed ALKBH7 was mainly correlated with inositol phosphate metabolism, small cell lung cancer, adheres junction, and dorsoventral axis formation pathways. HALLMARK terms demonstrated that low expression of ALKBH7 was associated with G2M checkpoint, TGF-β signaling, UV response, and mitotic spindle pathways. In the context of HNSC, GSEA revealed that ALKBH7 is linked to energy metabolism through oxidative phosphorylation and immune response via immune cytokine regulation (Figure 2E–2G). Over-representation analysis was also performed using GO and KEGG datasets in HNSC to exhibit ALKBH7-related biological functions systematically (Supplementary Figure 3C, 3D). Collectively, the above results indicated that ALKBH7 might be involved in the development and progression of tumors by affecting the metabolism and immune response, especially in HNSC.

### The immune microenvironment was indeed associated with ALKBH7 expression in multiple cancers

**Figure 3:**
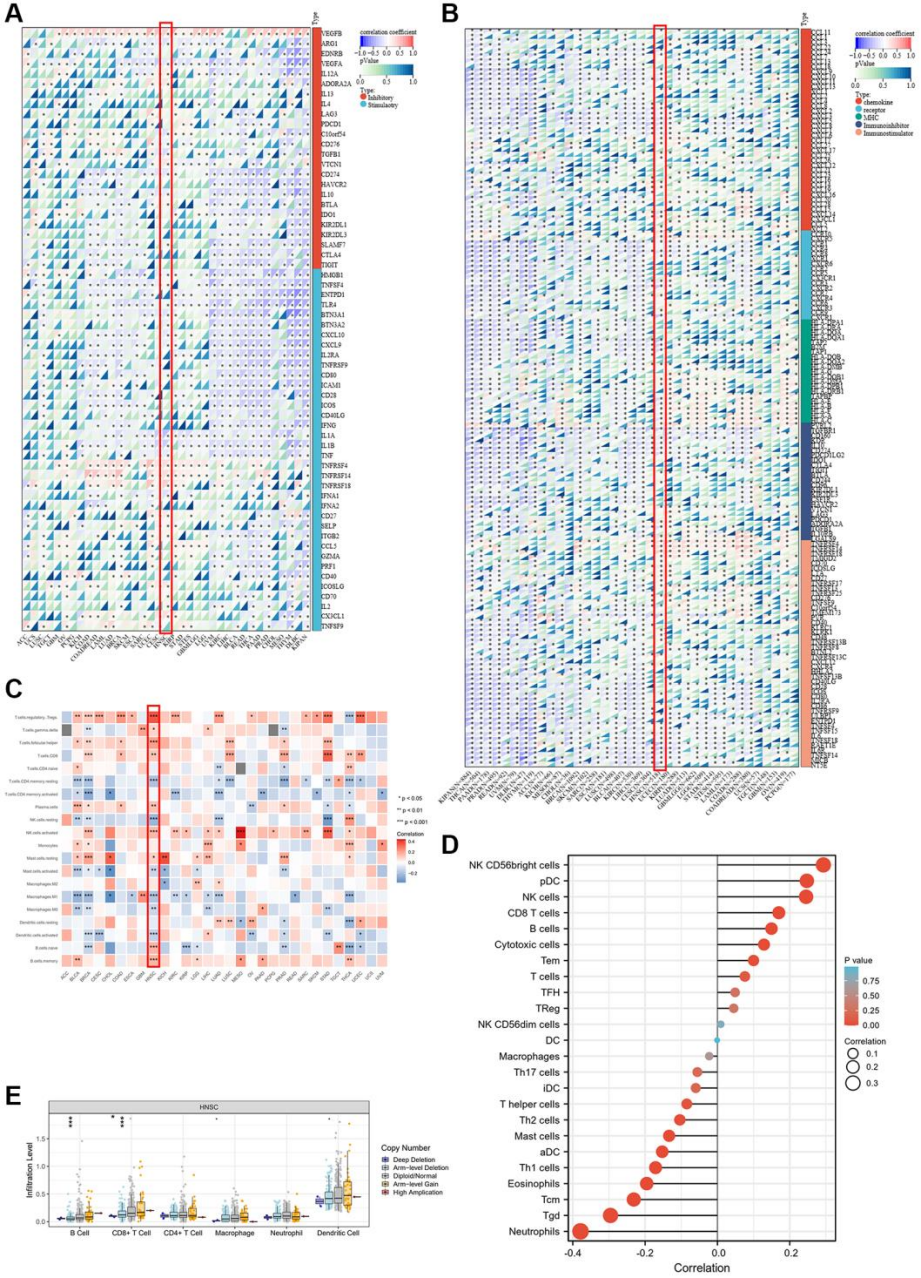
**Association between ALKBH7 expression and tumor immunity in pan-cancer.** (**A**) The correlation between ALKBH7 expression and immune check point related genes in different types of cancer. (**B**) The correlation between ALKBH7 expression and immunomodulator genes in different types of cancer. (**C**) The correlation between ALKBH7 expression and immune cell infiltration in different types of cancer. (**D**) Correlations between ALKBH7 expression and 24 immune cells in TCGA HNSC cohort. (**E**) Correlation of immune cell infiltrate level and different mutation subtypes based on TCGA HNSC cohort. ^*^p < 0.05, ^**^p < 0.01, ^***^p < 0.001.

Recently, the research on tumor microenvironments has been recognized more and more deeply, especially in the field of immune microenvironments. After analyzing the biological functions of ALKBH7 in Figure 2, we focused on the immune-related pathways. Firstly, we observed the association between ALKBH7 expression and the immune genes, including immunomodulator genes and immune checkpoint-related genes, which played essential roles in tumor immunotherapy for cancers (Figure 3A, 3B). Interestingly, our pooled analysis for immune inhibitory and stimulatory genes showed that ALKBH7 could play a negative regulatory role in several immune inhibitors of immune evasions, such as VEGFA, LAG3, PDCD1, CD274, HAVCR2, IL-10, IDO1, CTLA4 and TIGIT in BLCA, READ, THCA and KIPAN (Figure 3A). However, the regulatory relationship between immune inhibitory and stimulatory genes and ALKBH7 was found to be more complex in HNSC. Notably, genes like TNFRSF4, TNFRSF14, TNFRSF18, CX3CL1, and IL12A show a positive correlation with ALKBH7 expression, whereas genes like CD274, IL10, CXCL9, and CXCL10 exhibit a negative correlation with ALKBH7 expression. As shown in Figure 3B, the expression of ALKBH7 exerted a negative regulatory effect on immunomodulators, including chemokines, immune receptors, MHC, immune inhibitors, and immune stimulators in KIPAN, THCA, PAAD, and PRAD. Similarly, the expression of ALBH7 in HNSC was found to have a negative correlation with the majority of immunomodulators. The result indicated that ALKBH7 had the potential to impede the progression of various human tumors and boost immune surveillance through diverse immune oncological mechanisms. By analyzing well-recognized immune signatures, we further explored the relationship between ALKBH7 expression and immune cell infiltration across pan-cancer (Figure 3C). The finding revealed a positive correlation between ALKBH7 expression and Treg cells, activated NK cells, resting mast cells, and memory B cells. Conversely, a negative association was observed with CD4+ memory T cells and M1 macrophages. It was worth noting that the expression of ALKBH7 in HNSC was highly correlated with almost all kinds of immune cells, especially positively regulating Treg cells, follicular T helper cells, CD8+ T cells, actively NK cells, B cells, but negatively regulating resting NK cells, M1 macrophages, activated dendritic cells. Additionally, to verify the above results about quantitative analysis of infiltrating immunoreactive cells, various algorithms, including MCPcounter, EPIC, TIMER, and QUANTISEQ, were employed for statistics (Supplementary Figure 4A–4D).

To focus on immune infiltration in HNSC, a more detailed analysis of the immune infiltration in HNSC was performed based on the TCGA HNSC cohort (Figure 3D). The result showed a positive correlation between ALKBH7 expression and NK cells, pDCs, and CD8+ T cells. Conversely, ALKBH7 expression was negatively associated with neutrophils, central memory T cells, and γδT cells. Furthermore, we investigated the relationship between immune-related genomic aberrations and immune cell infiltration. Specifically, we found that the arm-level deletion of ALKBH7 significantly decreases the infiltration of CD8+ T cells and B cells, while the deep deletion of ALKBH7 cloud also leads to a reduction in CD8+ T cell infiltration (Figure 3E). In light of the immunosuppressive nature of the tumor microenvironment and the clinical heterogeneity, we utilized multiple datasets and algorithms to confirm the association between ALKBH7 and immunity in HNSC. The finding verified that ALKBH7 expression in HNSC might play a role in modulating immune cell infiltration, particularly in relation to T cell infiltration (Supplementary Figure 5A). In addition, our analysis indicated a positive correlation between ALKBH7 expression and most immunomodulators, including immune-stimulators and receptors (Supplementary Figure 5B). Consistently, ALKBH7 could regulate the tumor immune microenvironment through different mechanisms, especially in HNSC, which needed further meticulous experiments to decipher.

### The expression of ALKBH7 could roughly reflect the level of stemness, TMB, MSI, HRD, MMR deficiency, and DNA methyltransferases

**Figure 4:**
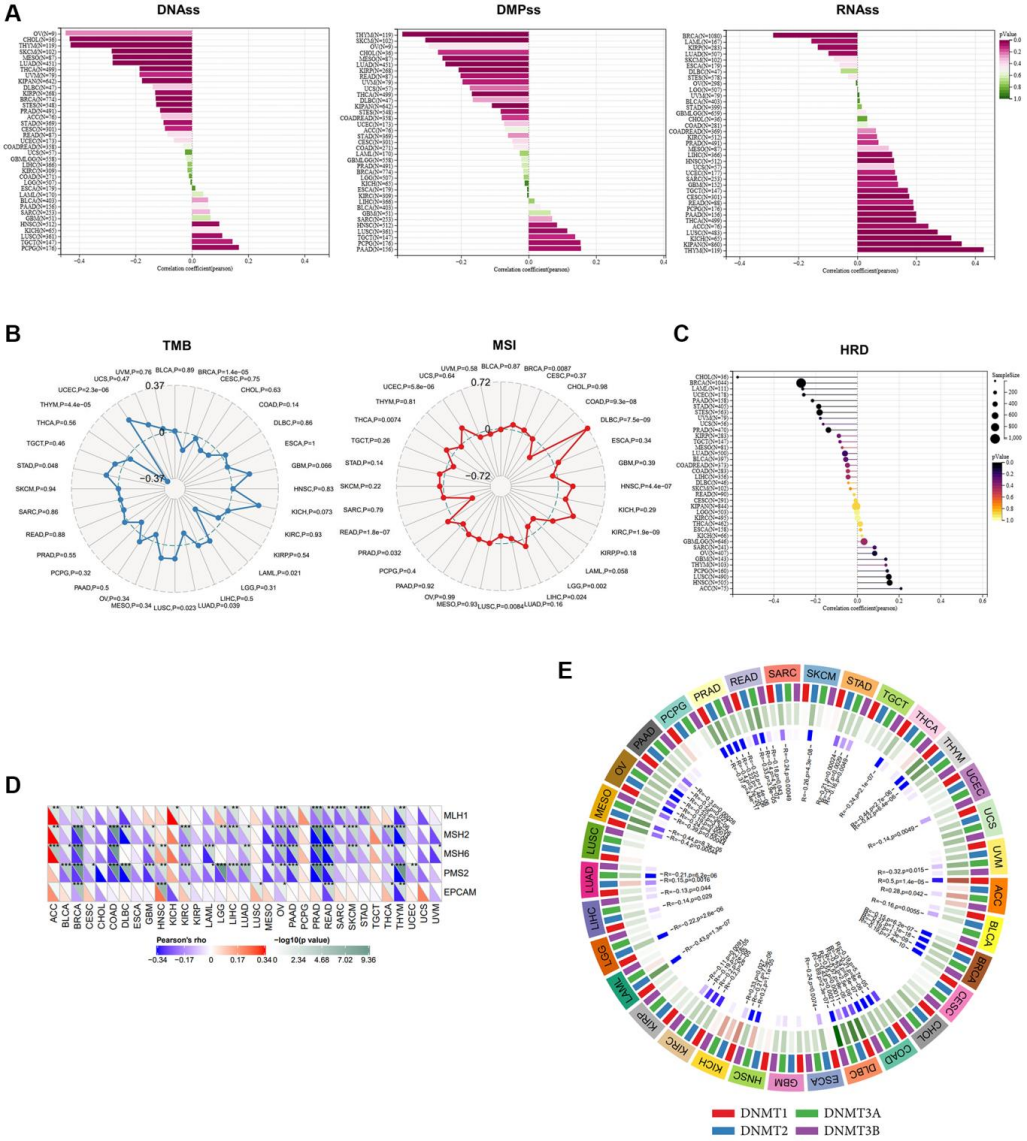
**The analysis of stemness, TMB, MSI, MMR deficiency and DNA methyltransferases in pan-cancer.** (**A**) Correlations of ALKBH7 expression with cancer stemness features based on DNAss, DMPss and RNAss (from left to right). (**B**) Correlations of ALKBH7 expression with TMB (left) and MSI (right). (**C**) Correlations of ALKBH7 expression with HRD. (**D**) Variation tendencies of 5 MMR genes. (**E**) Variation tendencies of 4 methyltransferases. ^*^p < 0.05, ^**^p < 0.01, ^***^p < 0.001.

Cancer stemness has been proven to be associated with prognosis and the efficacy of immunotherapy [24]. Consequently, we applied three stemness indices to delineate the correlation between ALKBH7 and stemness, including DNA methylation-based stemness scores (DNAss), differentially methylated probe-based stemness scores (DMPss), and RNA expression-based stemness scores (RNAss) (Figure 4A). ALKBH7 expression showed a negative correlation with DNAss and DMPss, and a positive correlation with RNAss across various cancers in pan-cancer analysis. Particularly in HNSC, LUSC, TGCT, and PCPG, a positive correlation was found between ALKBH7 expression and all three stemness indices. Tumor mutational burden (TMB) and microsatellite instability (MSI) have been recognized as predictors of immunotherapy in recent years [25]. As presented in our radar chart, ALKBH7 expression was positively correlated with the degree of TMB in UCEC but negatively correlated with TMB in THYM and LAML. Additionally, ALKBH7 expression displayed a positive correlation with MSI in 8 different cancer types such as DLBC, HNSC, and KIRC, but a negative correlation with MSI in READ, COAD, and LGG (Figure 4B). In addition to TMB and MSI, homologous recombination deficiency (HRD) and mismatch repair (MMR) related genes were also analyzed as the genome instability markers. A notable negative correlation was observed between ALKBH7 expression and HRD in CHOL, while a positive correlation was found in HNSC (Figure 4C). Five MMR genes were involved in our analysis, including MLH1, MSH2, MSH6, PMS2, and EPCAM, showing that ALKBH7 was negatively correlated with MMR genes in most cancers except several types of cancers such as ACC, HNSC, and KICH (Figure 4D). DNA methylation was reported to be a chemical modification form of DNA, which could significantly affect tumor microenvironment and immunotherapy [26]. The correlation between four methyltransferases and ALKBH7 was analyzed, showing in the circle visualization diagram (Figure 4E). The results showed that ALKBH7 expression might be negatively correlated with all four methyltransferases in 6 types of cancers, including OV, PRAD, BRCA, COAD, DLBC, and KIRC. In contrast, 7 types of cancers including PCPG, LUSC, KIRP, GBM, CHOL, CESC, UCS and TGCT were not associated with any methyltransferases. Remarkably, the expression level of ALKBH7 in HNSC was not related to the two methyltransferases (DNMT1 and DNMT2). However, it was positively correlated with DNMT3A and negatively correlated with DNMT3B, which were both important de novo DNA methyltransferases. Based on above findings, the expression of ALKBH7 might indirectly influence the response to immunotherapy in HNSC. In the other word, higher expression of ALKBH7 may indicate a higher response rate to therapy with immune checkpoint inhibitors.

### ALKBH7 expression could potentially affect drug sensitivity

**Figure 5:**
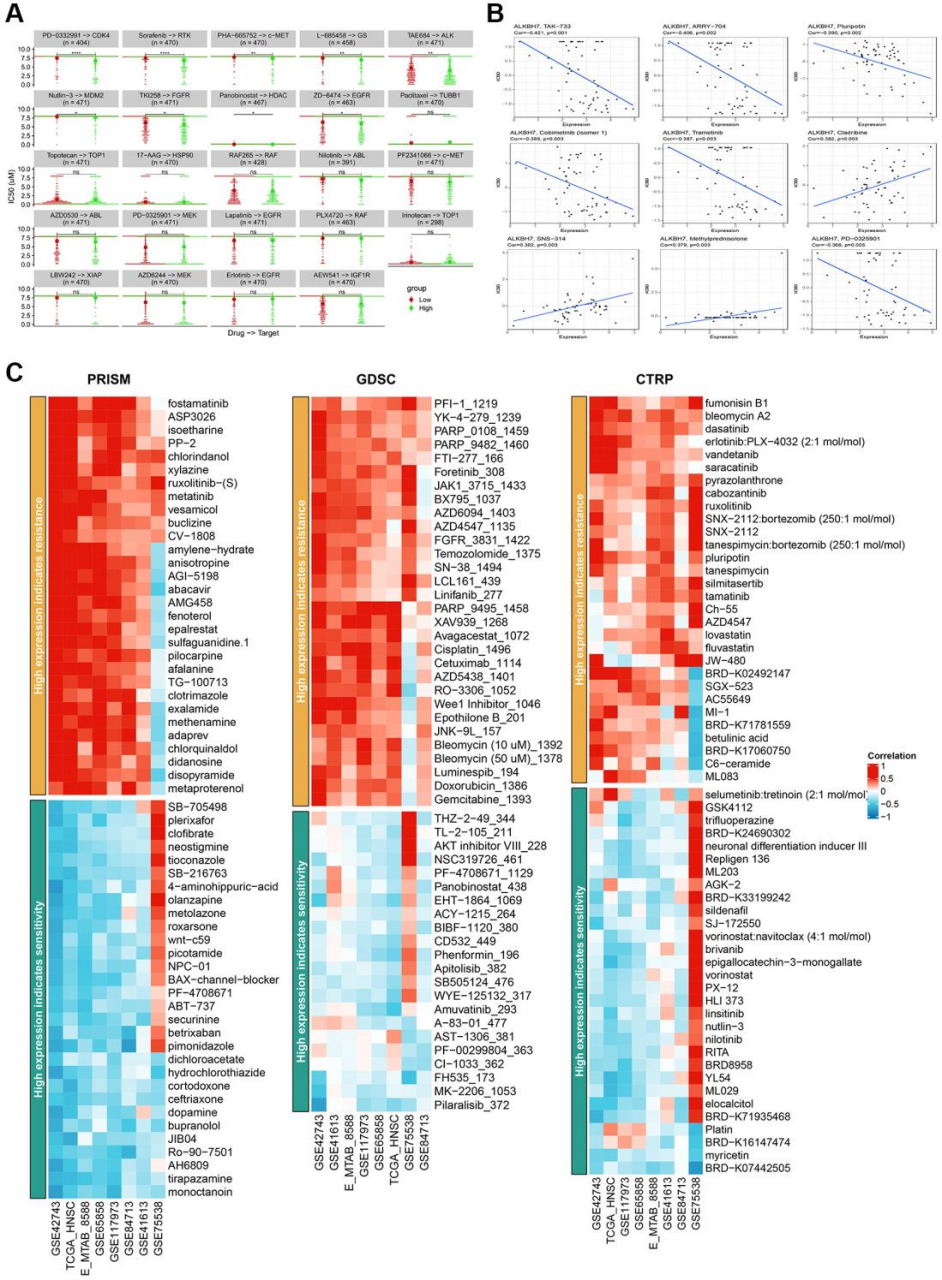
**Analysis of IC50 for different drugs in pan-cancer cell lines and drug sensitivity in HNSC from various databases.** (**A**) Comparison of IC_50_ of PD-0332991, Sorafenib, PHA-665752, L-685458, TAR684, Nutlin-3, TKI258, Panobinostat, ZD-6474, Paclitaxel, Topotecan, 17-AAG, RAF265, Nilotinib, PF2341066, AZD0530, PD-0325901, Lapatinib, PLX4720, Irinotecan, LBW242, AZD6244, Erlotinib and AEW541 between groups stratified by ALKBH7 expression levels. (**B**) Correlation between ALKBH7 expression and IC_50_ of TAK-733, ARRT-704, Pluripotin, Cobimetinib, Trametinib, Cladribine, SNS-314, Methylprednisolone and PD-0325901 in pan-cancer. (**C**) Correlation between the expression of ALKBH7 and drug sensitivity/resistance via 3 public drug sensitivity databases (PRISM, GDSC and CTRP). ^*^p < 0.05, ^**^p < 0.01, ^***^p < 0.001, ^****^p < 0.0001.

The development of drug resistance continues to be a major therapeutic hurdle, thus making the search for new potential targets and the regulation of drug sensitivity have always been the research highlights [27]. To find out the correlation between ALKBH7 expression and drug sensitivity in pan-cancer, we collected IC_50_ data from Cell Miner and GDSC databases. It is obvious that IC_50_ of PD−0332991, Sorafenib, PHA-665752, L-685458, TAR684, Nutlin-3, TKI258, and ZD-6474 in the high-expressed ALKBH7 group were significantly lower than those in low-expressed ALKBH7 group (Figure 5A). Besides, the expression of ALKBH7 was inversely correlated with the targeted drugs TAK−733, ARRY-704, Pluripotin, Cobimetinib (isomer1), Trametinib, PD-0325901. Conversely, the expression of ALKBH7 was positively associated with the targeted drugs Cladribine, SNS-314, and Methylprednisolone (Figure 5B). Subsequently, we analyzed the correlation between ALKBH7 expression and drug sensitivity or resistance in HNSC based on three public drug sensitivity databases, including PRISM, GDSC, and CTRP (Figure 5C). These results indicated that the expression of ALKBH7 might provide a new basis for drug selection of multiple cancers, especially of HNSC.

### ALKBH7 expression could inhibit cell proliferation and invasion in LSC-1 cells

**Figure 6:**
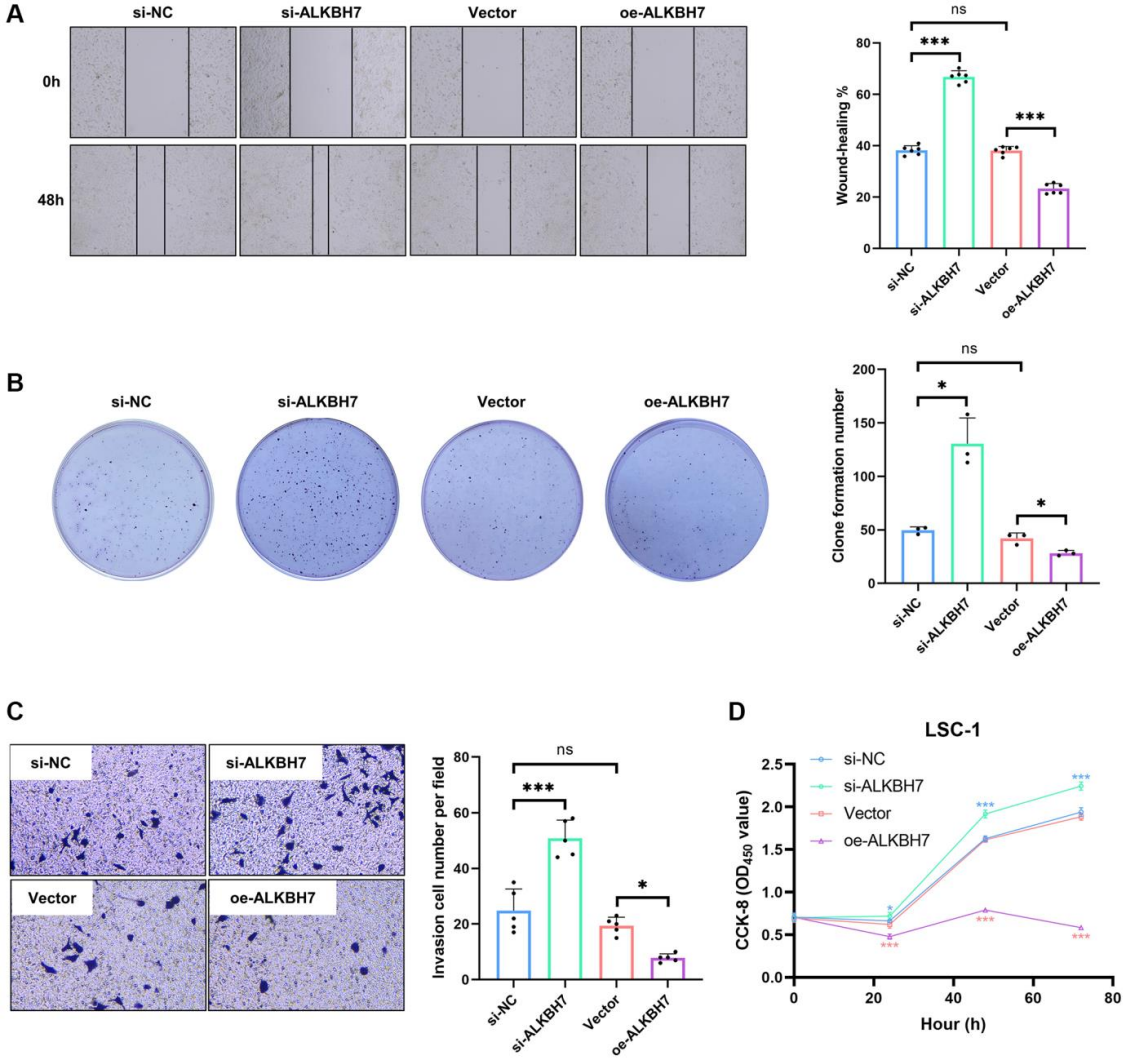
**Effect of ALKBH7 expression on the function of LSC-1.** (**A**) The wound healing assay of LSC-1 after stably up/down-regulating ALKBH7 expression, *n* = 6. (**B**) The cell colony formation assay of LSC-1 after stably up/down-regulating ALKBH7 expression, *n* = 3. (**C**) The transwell invasion assay of LSC-1 after stably up/down-regulating ALKBH7 expression, *n* = 5. (**D**) The cell proliferation of LSC-1 after stably up/down-regulating ALKBH7 expression, *n* = 6. ^*^p < 0.05, ^**^p < 0.01, ^***^p < 0.001.

The laryngeal squamous cell carcinoma cell line (LSC-1) was one of the typical cell lines of HNSC *in vitro*, established from Chinese populations [28]. We firstly knocked down or overexpressed ALKBH7 in LSC-1 via transfection of siRNA and plasmid (Supplementary Figure 6A–6C), showing that knockdown of ALKBH7 could significantly increase the ability of cell migration. In contrast, overexpressed ALKBH7 exerted an opposite effect (Figure 6A). Subsequently, the colony formation assay and the transwell invasion assay were performed to compare cell proliferation and invasion function. The result showed that the knockdown of ALKBH7 could increase the number of colon formations and the invasive number, while overexpressed ALKBH7 decreased (Figure 6B, 6C). Finally, we observed the cell proliferating activity using cell counting kit-8 (CCK-8), suggesting that down-regulating ALKBH7 expression could improve the activity of cell proliferation at the indicated times (24 h, 48 h, and 72 h) (Figure 6D). Collectively, the results of *in vitro* experiments showed a potential protective effect of ALKBH7 in HNSC. However, further experimental analysis is needed to elucidate the specific molecular mechanisms involved.

## DISCUSSION

Pan-cancer analysis can provide insights into cancer development, immune cell infiltration, and potential therapeutic drug screening while revealing tumor differences [29, 30]. Although previous analysis for has confirmed the prognostic value of ALKBH7 in some cancers [22, 23], we performed a more detailed and comprehensive bioinformatics analysis to determine its impact on multiple cancer types, especially on HNSC.

Our pan-cancer analysis revealed a significant reduction in ALKBH7 expression in various tumor tissues compared to normal tissues, especially in HNSC. Notably, ALKBH7 was the few genes in the ALKBH family to exhibit significant reduction, highlighting the importance of exploring its expression and function in HNSC. The findings showed that ALKBH7 mRNA expression was down-regulated in seven cancer types (COAD, HNSC, KIRC, LUAD, STAD, THCA, and UCEC) and up-regulated in only four tumors (BRCA, KICH, LIHC, and PRAD), in consistent with a previous study [23]. By performing RNA m1A, m5C, and m6A methylation and DNA methylation analysis, we found that ALKBH7 was negatively correlated with RNA modification-related genes and DNA methylation in most cancers, and the exact mechanism needs further investigation. The Kaplan-Meier OS analysis showed that survival was worse in cancer patients with reduced ALKBH7 expression, especially in the laryngeal squamous cell carcinoma, suggesting that low ALKBH7 expression is associated with poor survival outcomes in HNSC.

The specific mechanism through which ALKBH7 contributes to the development and advancement of tumors is still not fully understood. We indicated that ALKBH7 negatively regulates oncogenic pathways such as MTORC1 signaling, KRAS signaling, G2M checkpoint, and mitotic spindle pathway by functional enrichment analysis. Notably, ALKBH7 was significantly associated with energy metabolism-related pathways such as oxidative phosphorylation and fatty acid metabolism, consistent with previous studies [12, 13]. Limitless replicative potential and deregulating cellular energetics are thought to be closely associated with tumor metastasis, immune checkpoint blockade therapies, and immunotherapy resistance [31–35]. These results suggest that ALKBH7 may inhibit tumorigenesis and progression by affecting multiple pathways, including oncogenic pathways and energy metabolism.

Immune checkpoints consist of stimulatory and suppressive pathways and are essential immunomodulators for maintaining immune homeostasis [36]. We found that ALKBH7 was negatively correlated with most immune checkpoint-related genes in pan-cancer. In addition, correlation analysis of ALKBH7 with pan-cancer immune regulators showed that ALKBH7 expression was highly negatively correlated with the expression of chemokines, immune receptors, MHC, immunosuppressants, and immune activators. The tumor immune microenvironment is likely a key determinant of sensitivity to immune checkpoint inhibitor treatment, and analysis of signaling and crosstalk between immune cells and tumor cells could help us to understand more about the mechanisms of tumor immune evasion [37, 38]. Another important finding is that ALKBH7 expression is highly correlated with immune infiltration of cancer and that ALKBH7 expression is significantly correlated with NK cells, pDCs, CD8+ T cells, neutrophils, central memory T cells, and γδ T cells. Genomic abnormalities in ALKBH7 significantly reduced CD8+ T-cell immune infiltration, while CD8+ T-cell depletion significantly reduced immune surveillance capacity and the ability to clear tumor cells [39–41]. These results suggest that ALKBH7 is most likely involved in cancer progression and prognosis by interacting with the cancer microenvironment.

TMB and MSI can be sensitive predictors of immune checkpoint inhibitor efficacy [42], and ALKBH7 expression was associated with TMB in 7 cancer types and MSI in 12 cancer types. HRD and MMR are essential factors in assessing genome stability and are closely related to immune response [43–45]. Except for ACC, HNSC, and KICH, ALKBH7 expression was significantly negatively correlated with the expression of five MMR genes in pan-cancer, which is also negatively correlated with all methyl transporter enzymes in multiple cancer types. These findings imply that ALKBH7 expression may reshape the immunosuppressive microenvironment in pan-cancer by regulating the epigenetic status of tumors.

Based on Cell Miner and GDSC data, the analysis of IC_50_ values of different drugs in pan-cancer reveals that high ALKBH7 expression might be sensitive to most anti-cancer drugs, such as Palbociclib, Dovitinib, Vandetanib, Trametinib, Mirdametinib, and Pluripotin. In addition, drug sensitivity analysis of 172 anticancer drugs in HNSC based on three databases indicated that HNSC patients with high ALKBH7 expression were beneficial and less likely to develop resistance to most anticancer drug treatments, thus making ALKBH7 might serve as a potential biological indicator for drug resistance testing. Current studies showed that platum agents were the cornerstone of chemotherapy for HNSC, but plagued with high risk of drug resistance and toxic side effect [46]. Our analysis showed that high expression of ALKBH7 in HNSC might lead to resistance to cisplatin based on GDSC data, but it might also increase sensitivity to platum agents based on CTRP data. Therefore, for patients with high expression of ALKBH7, we could consider choosing to use low toxicity platinum drugs with mild efficacy for chemotherapy.

Since the conclusions are based on bioinformatics analysis of TCGA or GEO datasets, we validated the bioinformatics analysis by overexpressing and silencing the expression of ALKBH7, showing that LSC-1 cells overexpressing ALKBH7 have restricted cell proliferation, migration, and invasion. Nevertheless, our study has some limitations. First, our study assessed the correlation between the mRNA and protein levels of ALKBH7 and the development and progression of laryngeal squamous cell carcinoma, a highly prevalent cancer in HNSC. However, it is important to emphasize that this association should be further investigated in other types of cancer in future studies. Second, since public datasets are continuously updated, our findings will require additional validation in newer and more extensive public datasets in the future. Third, it is essential to acknowledge that our study relied on the analysis of multiple datasets. The information processing, data analysis, and update times of these datasets may vary, potentially introducing systemic bias. Therefore, it is imperative to conduct further mechanistic research to explore the role of ALKBH7 in cancer tumorigenesis and progression and to assess its value of potential as an anticancer therapeutic target.

## CONCLUSIONS

We conducted a thorough evaluation of ALKBH7 and discovered its potential as a tumor suppressor, as well as its significance as a prognostic indicator for patients. Notably, increased expression of ALKBH7 may have a protective effect on the development and progression of various cancers, particularly HNSC, by influencing metabolism and immune cell infiltration. Consequently, ALKBH7 expression could serve as a novel biomarker and immunotherapeutic target for future prognostic purposes.

## MATERIALS AND METHODS

### Data collection

Normalized expression profile data, TMB data, MSI data, and clinical information of pan-cancer in TCGA datasets were collected from UCSC Xena (https://xena.ucsc.edu) [47, 48]. GTEx (https://commonfund.nih.gov/GTEx) was employed to obtain the gene expression data of normal tissues. The abbreviations and corresponding full names of various cancers were shown in Supplementary Table 2. CCLE (https://sites.broadinstitute.org/ccle) was adopted to acquire the expression data of different cancer cell lines. The outhouse datasets for HNSC (including GSE84713, GSE117973, GSE65858, GSE41613, GSE42743, GSE75538, and E_MTAB_8588) were used to specifically verify the relevant conclusions of ALKBH7 in pan-caner analysis. The RNA-seq read counts collected from public databases were converted to TPM. As datasets came from public databases, institutional review board approval and informed consent were not required. Patients were excluded if they 1) did not have prognostic information and 2) died in 30 days.

### Differential expression analysis

Analysis of ALKBH7 expression in pan-cancer, especially in HNSC, was performed based on TPM format RNAseq data processed uniformly by the Toil process in TCGA and GTEx database, using *p*-value < 0.05 and absolute fold change >1.5 as the threshold [48].

### Survival analysis

Prognosis indicators, including OS, DSS, DFI, and PFI, were analyzed to assess the role of ALKBH7 in tumor prognosis using univariate Cox regression analysis. The Kaplan-Meier curves were used to focus on the impact of ALKBH7 expression on OS in HNSC using the log-rank test. A *p*-value < 0.05 was considered statistically significant.

### Enrichment analysis

Pearson’s correlation between ALKBH7 and other mRNA retrieved from the TCGA database was analyzed to sort the genes through the level of correlation index. The genes most correlated with ALKBH7 expression were selected for enrichment analysis. R package “cluster profiler” was employed to perform Gene Ontology (GO) analysis, Kyoto Encyclopedia of Genes and Genomes (KEGG) analysis and Gene Set Enrichment Analysis (GSEA), and R package “gsva” was employed to perform Gene Set Variation Analysis (GSVA) [49, 50].

### Evaluation of immune signature and immune cell infiltration

The co-expression analysis of immune-related genes (including immune checkpoint-related genes and immunomodulator genes) and ALKBH7 was performed via R package “limma”, “reshape2”, and “RColorBrewer”. R package “CIBERSORT” was employed to quantify the immune cell infiltration, and then four algorithms (including MCPcounter, EPIC, TIMER and QUANTISEQ) were applied to validate the quantification among pan-cancer [51]. We also used ssGSEA algorithm in R package “gsva” to analyze the correlation of immune cell infiltration and ALKBH7 expression level in HNSC [50]. The TIMER2.0 website (http://timer.cistrome.org/) was employed to validate the effect of ALKBH7 mutation on immune cell infiltration in HNSC [52].

### Evaluation of genomic alteration

The genome instability markers were analyzed, which included homologous recombination deficiency (HRD), Tumor mutational burden (TMB), microsatellite instability (MSI), and mutational mismatch repair (MMR). We also evaluated the landscapes of DNA methylation methyltransferases (DNMT), including DNMT1, DNMT2, DNMT3A, and DNMT3B. All these datasets were collected from the TCGA database, and the analysis was based on Spearman’s method.

### Analysis of potential chemotherapy responses

The correlation between ALKBH7 expression and chemotherapy response was analyzed based on the data of half maximal inhibitory concentration (IC_50_) and gene expression of cancer cell lines, which was respectively collected from Cell Miner (https://discover.nci.nih.gov/cellminer/home.do) and GDSC (https://www.cancerrxgene.org/) database [53, 54]. In addition, the outhouse datasets for HNSC were used to validate the correlation of drug sensitivity or resistance with ALKBH7 expression based on PRISM, GDSC, and CTRP databases.

### Samples collection

30 tissue samples (15 tumor tissues and 15 normal tissues surrounding the tumor) from 15 patients (10 male and 5 females; age range, 44–65 years) with HNSC involved in this study were from the Department of Otolaryngology Head and Neck Surgery, Second Affiliated Hospital of Naval Medical University, Shanghai, China. All samples were collected and stored in liquid nitrogen during radical resection of HNSC.

### Immunohistochemical staining

The tissue sections with a thickness of 4 μm were cut from 4% paraformaldehyde-fixed and paraffin-embedded tissues using routine methods. After de-waxed with xylene and rehydrated, the sections were retrieved in a pressure cooker for 3 min at pH 6.0 in 10 mM citrate buffer and then blocked at room temperature using TBS solution containing 10% normal serum and 1% BSA for 2 h. The sections were incubated with primary antibody (Anti-ALKBH7, 1:50, ab204568, Abcam, UK) overnight at 4°C. Endogenous peroxidase activity was quenched in TBS solution containing 0.3% H_2_O_2_ for 15 min. The immunostaining was performed using Dako ChemMate EnVision Detection Kit Peroxidase/Diaminobenzidine (DAB) Rabbit/Mouse (K500711-2CN, Dako, Denmark), which resulted in a brown-colored precipitate at the antigen site. Subsequently, sections were counterstained with hematoxylin (ab220365, Abcam), mounted in a non-aqueous mounting medium, and cover slipped. The H-Score of each image was automatically calculated by Qunant Center 2.1 using a formula “ HScore=∑(PI×I). *PI*, the percentage of positive signal pixel area; *I*, the staining intensity. (0 = no staining, 1 = weak, 2 = moderate, 3 = strong)”.

### Western blotting

Proteins were separated using a 10% SDS-PAGE gel and then transferred to PVDF membranes. After blocking in 5% bovine serum albumin (BSA), the membrane was probed with the primary antibodies (Anti-ALKBH7, 1:100, ab204568, Abcam; Anti-β-actin, 1:5000, ab8227, Abcam) and developed with an HRP-linked secondary antibody (Goat anti-Rabbit IgG, 1:5000, 111-035-003, Jackson) using enhanced chemiluminescence.

### qPCR

Total RNA was extracted from tissue samples using the classic TRIzol method and reverse transcribed into cDNA following the protocol of the ReverTra Ace qPCR RT Master Mix (FSQ-201, Toyobo, Japan). The 2 × Universal Blue SYBR Green qPCR Master Mix (G3326-15, Servicebio, China) instruction manual was followed for cDNA dilution and detection. Gapdh was utilized as the internal reference for data processing, and the 2^−ΔΔCT^ method was employed to calculate the relative expression levels of various target mRNAs. The primer sequences for target genes were as follows: Human Gapdh F/R, ACAACTTTGGTATCGTGGAA GG/GCCATCACGCCACAGTTTC; Human ALKBH7 F/R, GACCCCGCTCCGGGATTATG/TCTGTCTCTC GGAAGCCGT.

### Cell culture and transfection

The normal human laryngeal squamous cell carcinoma cell line LSC-1 was purchased from the Shanghai Cell Bank of Bluefcell (BFN60810341) and cultured in 5% CO_2_ at 37°C in DMEM (Gibco, USA) containing 10% FBS (Gibco) and 1% Penicillin-Streptomycin (Gibco). The cells were evaluated in the logarithmic growth phase. A siRNA against ALKBH7 (si-ALKBH7) and pcDNA3.1(+)-ALKBH7 (oe-ALKBH7, GS1-22040178, Gene Create) were purchased, and the transfection was performed using Lipo6000^™^ Transfection Reagent (Beyotime, China).

### Cell counting kit-8 (CCK-8)-based cell proliferation assay

LSC-1 cells were seeded in a 96-well plate with a density of 5 × 10^4^ cells per well to assess cell proliferating activity. At the indicated times after transfection (0, 24, 48, and 72 h), 10 μL CCK-8 solution (Beyotime) was added to each well, and the plates were incubated at 37°C for 3 h. The cell proliferation was determined with a CCK-8 assay kit (Beyotime). The absorbance was measured at 450 nm.

### Wound healing assay

The LSC-1 cells were seeded in 6-well plates and incubated to nearly 100% confluence. The cell monolayer was scratched with a 10 μL plastic pipette tip along the diameter of the well. After discarding the cell culture medium, the wells were washed with phosphate-buffered saline (PBS), 4% paraformaldehyde (PFA) was added to the well, and the area of scratch closure was used to estimate the migratory ability with an inverted phase microscope. The image data were analyzed by ImageJ 1.45, and the percentage of wound closure was calculated as a ratio of the difference between the wound area at 0 h and 48 h to the wound area at 0 h.

### Cell colony formation

The LSC-1 cells (5000 cells/2 mL) were plated into 3.5 cm cell culture dishes and incubated for two weeks. The colonies were fixed with 4% PFA and stained with 1% crystal violet. The colony formation numbers were counted with an inverted phase microscope.

### Transwell invasion assay

The cell invasion was detected by the transwell assay. The transwell plates were pretreated by Matrigel (dissolved in serum-free DMEM at a ratio of 1/3). The DMEM with 10% FBS was added to the lower chamber, and 5 × 10^4^ cells in 500 μL serum-free DMEM were added to the upper chambers. After incubation for 24 h at 37°C with 5% CO_2_, the upper surface of the membrane was wiped with a cotton tip, and the cells on the lower membrane were fixed with methanol for 15 min and then stained with 0.1% crystal violet for 30 min. The invasive cell numbers per field were counted with an inverted phase microscope.

### Statistical analysis

R software (version 4.0.4) was used for data processing, statistical analysis, and plotting. Differences in ALKBH7 expression in the public datasets were compared by one-way ANOVA. Differences in OS, DSS, PFI, and DFI between two subgroups were compared by the Kaplan-Meier method and log-rank test, and the hazard ratios (HRs) were obtained by univariate Cox regression and multiple Cox regression analysis. Correlation analysis was performed using Pearson’s correlation test. All the quantification of images were analyzed using ImageJ Software (version 1.45). All experimental data were expressed as mean ± SEM and analyzed using Graph Pad software (version 8.0) with *t*-test between two groups and one-way ANOVA for three or more groups. All *p*-values were two-sided, with *p* < 0.05 as statistically significant.

### Data availability statement

The original contributions presented in the study are included in the article/Supplementary Material; further inquiries can be directed to the corresponding author.

## Supplementary Materials

Supplementary Figures

Supplementary Tables

## References

[1] Johnson DE, Burtness B, Leemans CR, Lui VWY, Bauman JE, Grandis JR. Head and neck squamous cell carcinoma. Nat Rev Dis Primers. 2020; 6:92. 10.1038/s41572-020-00224-3 33243986 PMC7944998

[2] Sánchez-Danés A, Blanpain C. Deciphering the cells of origin of squamous cell carcinomas. Nat Rev Cancer. 2018; 18:549–61. 10.1038/s41568-018-0024-5 29849070 PMC7170720

[3] Ferlay J, Colombet M, Soerjomataram I, Mathers C, Parkin DM, Piñeros M, Znaor A, Bray F. Estimating the global cancer incidence and mortality in 2018: GLOBOCAN sources and methods. Int J Cancer. 2019; 144:1941–53. 10.1002/ijc.31937 30350310

[4] Bray F, Ferlay J, Soerjomataram I, Siegel RL, Torre LA, Jemal A. Global cancer statistics 2018: GLOBOCAN estimates of incidence and mortality worldwide for 36 cancers in 185 countries. CA Cancer J Clin. 2018; 68:394–424. 10.3322/caac.21492 30207593

[5] Cancer Genome Atlas Network. Comprehensive genomic characterization of head and neck squamous cell carcinomas. Nature. 2015; 517:576–82. 10.1038/nature14129 25631445 PMC4311405

[6] Seiwert TY, Burtness B, Mehra R, Weiss J, Berger R, Eder JP, Heath K, McClanahan T, Lunceford J, Gause C, Cheng JD, Chow LQ. Safety and clinical activity of pembrolizumab for treatment of recurrent or metastatic squamous cell carcinoma of the head and neck (KEYNOTE-012): an open-label, multicentre, phase 1b trial. Lancet Oncol. 2016; 17:956–65. 10.1016/S1470-2045(16)30066-3 27247226

[7] Burtness B, Harrington KJ, Greil R, Soulières D, Tahara M, de Castro G Jr, Psyrri A, Basté N, Neupane P, Bratland Å, Fuereder T, Hughes BGM, Mesía R, et al, and KEYNOTE-048 Investigators. Pembrolizumab alone or with chemotherapy versus cetuximab with chemotherapy for recurrent or metastatic squamous cell carcinoma of the head and neck (KEYNOTE-048): a randomised, open-label, phase 3 study. Lancet. 2019; 394:1915–28. 10.1016/S0140-6736(19)32591-7 31679945

[8] Borcoman E, Marret G, Le Tourneau C. Paradigm Change in First-Line Treatment of Recurrent and/or Metastatic Head and Neck Squamous Cell Carcinoma. Cancers (Basel). 2021; 13:2573. 10.3390/cancers13112573 34073885 PMC8197177

[9] Sacco AG, Chen R, Worden FP, Wong DJL, Adkins D, Swiecicki P, Chai-Ho W, Oppelt P, Ghosh D, Bykowski J, Molinolo A, Pittman E, Estrada MV, et al. Pembrolizumab plus cetuximab in patients with recurrent or metastatic head and neck squamous cell carcinoma: an open-label, multi-arm, non-randomised, multicentre, phase 2 trial. Lancet Oncol. 2021; 22:883–92. 10.1016/S1470-2045(21)00136-4 33989559 PMC12140401

[10] Zhang LS, Xiong QP, Peña Perez S, Liu C, Wei J, Le C, Zhang L, Harada BT, Dai Q, Feng X, Hao Z, Wang Y, Dong X, et al. ALKBH7-mediated demethylation regulates mitochondrial polycistronic RNA processing. Nat Cell Biol. 2021; 23:684–91. 10.1038/s41556-021-00709-7 34253897 PMC8716185

[11] Williams SC, Austin RN. An Overview of the Electron-Transfer Proteins That Activate Alkane Monooxygenase (AlkB). Front Microbiol. 2022; 13:845551. 10.3389/fmicb.2022.845551 35295299 PMC8918992

[12] Fu D, Jordan JJ, Samson LD. Human ALKBH7 is required for alkylation and oxidation-induced programmed necrosis. Genes Dev. 2013; 27:1089–100. 10.1101/gad.215533.113 23666923 PMC3672644

[13] Wang G, He Q, Feng C, Liu Y, Deng Z, Qi X, Wu W, Mei P, Chen Z. The atomic resolution structure of human AlkB homolog 7 (ALKBH7), a key protein for programmed necrosis and fat metabolism. J Biol Chem. 2014; 289:27924–36. 10.1074/jbc.M114.590505 25122757 PMC4183825

[14] Fischer J, Koch L, Emmerling C, Vierkotten J, Peters T, Brüning JC, Rüther U. Inactivation of the Fto gene protects from obesity. Nature. 2009; 458:894–8. 10.1038/nature07848 19234441

[15] Meng S, Zhan S, Dou W, Ge W. The interactome and proteomic responses of ALKBH7 in cell lines by in-depth proteomics analysis. Proteome Sci. 2019; 17:8. 10.1186/s12953-019-0156-x 31889914 PMC6935500

[16] Cai Y, Wu G, Peng B, Li J, Zeng S, Yan Y, Xu Z. Expression and molecular profiles of the AlkB family in ovarian serous carcinoma. Aging (Albany NY). 2021; 13:9679–92. 10.18632/aging.202716 33744868 PMC8064172

[17] Wu G, Yan Y, Cai Y, Peng B, Li J, Huang J, Xu Z, Zhou J. ALKBH1-8 and FTO: Potential Therapeutic Targets and Prognostic Biomarkers in Lung Adenocarcinoma Pathogenesis. Front Cell Dev Biol. 2021; 9:633927. 10.3389/fcell.2021.633927 34150745 PMC8209387

[18] Peng B, Yan Y, Xu Z. The bioinformatics and experimental analysis of AlkB family for prognosis and immune cell infiltration in hepatocellular carcinoma. PeerJ. 2021; 9:e12123. 10.7717/peerj.12123 34557360 PMC8418211

[19] Hayashi C, Takagi K, Sato A, Yamaguchi M, Minemura H, Miki Y, Harada-Shoji N, Miyashita M, Sasano H, Suzuki T. D-2-hydroxyglutarate dehydrogenase in breast carcinoma as a potent prognostic marker associated with proliferation. Histol Histopathol. 2021; 36:1053–62. 10.14670/HH-18-362 34296423

[20] Wang L, Feng X, Jiao Z, Gan J, Meng Q. Characterization of the prognostic and diagnostic values of ALKBH family members in non-small cell lung cancer. Pathol Res Pract. 2022; 231:153809. 10.1016/j.prp.2022.153809 35180653

[21] Feng S, Xu Z, Peng J, Zhang M. The AlkB Family: Potential Prognostic Biomarkers and Therapeutic Targets in Glioblastoma. Front Oncol. 2022; 12:847821. 10.3389/fonc.2022.847821 35371987 PMC8965608

[22] Chang WH, Forde D, Lai AG. Dual prognostic role of 2-oxoglutarate-dependent oxygenases in ten cancer types: implications for cell cycle regulation and cell adhesion maintenance. Cancer Commun (Lond). 2019; 39:23. 10.1186/s40880-019-0369-5 31036064 PMC6489267

[23] Chen K, Shen D, Tan L, Lai D, Han Y, Gu Y, Lu C, Gu X. A Pan-Cancer Analysis Reveals the Prognostic and Immunotherapeutic Value of ALKBH7. Front Genet. 2022; 13:822261. 10.3389/fgene.2022.822261 35222541 PMC8873580

[24] Shi X, Liu Y, Cheng S, Hu H, Zhang J, Wei M, Zhao L, Xin S. Cancer Stemness Associated With Prognosis and the Efficacy of Immunotherapy in Adrenocortical Carcinoma. Front Oncol. 2021; 11:651622. 10.3389/fonc.2021.651622 34367952 PMC8334864

[25] Georgiadis A, Durham JN, Keefer LA, Bartlett BR, Zielonka M, Murphy D, White JR, Lu S, Verner EL, Ruan F, Riley D, Anders RA, Gedvilaite E, et al. Noninvasive Detection of Microsatellite Instability and High Tumor Mutation Burden in Cancer Patients Treated with PD-1 Blockade. Clin Cancer Res. 2019; 25:7024–34. 10.1158/1078-0432.CCR-19-1372 31506389 PMC6892397

[26] Chakravarthy A, Furness A, Joshi K, Ghorani E, Ford K, Ward MJ, King EV, Lechner M, Marafioti T, Quezada SA, Thomas GJ, Feber A, Fenton TR. Pan-cancer deconvolution of tumour composition using DNA methylation. Nat Commun. 2018; 9:3220. 10.1038/s41467-018-05570-1 30104673 PMC6089972

[27] Pan R, Ruvolo V, Mu H, Leverson JD, Nichols G, Reed JC, Konopleva M, Andreeff M. Synthetic Lethality of Combined Bcl-2 Inhibition and p53 Activation in AML: Mechanisms and Superior Antileukemic Efficacy. Cancer Cell. 2017; 32:748–60.e6. 10.1016/j.ccell.2017.11.003 29232553 PMC5730338

[28] Wu CP, Zhou L, Gong HL, Du HD, Tian J, Sun S, Li JY. Establishment and characterization of a novel HPV-negative laryngeal squamous cell carcinoma cell line, FD-LSC-1, with missense and nonsense mutations of TP53 in the DNA-binding domain. Cancer Lett. 2014; 342:92–103. 10.1016/j.canlet.2013.08.041 24001612

[29] Combes AJ, Samad B, Tsui J, Chew NW, Yan P, Reeder GC, Kushnoor D, Shen A, Davidson B, Barczak AJ, Adkisson M, Edwards A, Naser M, et al, and Immunoprofiler Consortium. Discovering dominant tumor immune archetypes in a pan-cancer census. Cell. 2022; 185:184–203.e19. 10.1016/j.cell.2021.12.004 34963056 PMC8862608

[30] Chen R, Wu W, Chen SY, Liu ZZ, Wen ZP, Yu J, Zhang LB, Liu Z, Zhang J, Luo P, Zeng WJ, Cheng Q. A Pan-Cancer Analysis Reveals CLEC5A as a Biomarker for Cancer Immunity and Prognosis. Front Immunol. 2022; 13:831542. 10.3389/fimmu.2022.831542 35979347 PMC9376251

[31] Hanahan D. Hallmarks of Cancer: New Dimensions. Cancer Discov. 2022; 12:31–46. 10.1158/2159-8290.CD-21-1059 35022204

[32] Liu Y, Li H, Wilson CN, Bai HJ, Boufraqech M, Weyemi U. Histone H2AX promotes metastatic progression by preserving glycolysis via hexokinase-2. Sci Rep. 2022; 12:3758. 10.1038/s41598-022-07675-6 35260660 PMC8904825

[33] Hahn AW, Menk AV, Rivadeneira DB, Augustin RC, Xu M, Li J, Wu X, Mishra AK, Gide TN, Quek C, Zang Y, Spencer CN, Menzies AM, et al. Obesity Is Associated with Altered Tumor Metabolism in Metastatic Melanoma. Clin Cancer Res. 2023; 29:154–64. 10.1158/1078-0432.CCR-22-2661 36166093 PMC10311539

[34] Guerra L, Bonetti L, Brenner D. Metabolic Modulation of Immunity: A New Concept in Cancer Immunotherapy. Cell Rep. 2020; 32:107848. 10.1016/j.celrep.2020.107848 32640218

[35] Liu L, Liang L, Mai G, Chen Y. A novel fatty acid metabolism-related gene signature predicts the prognosis, tumor immune properties, and immunotherapy response of colon adenocarcinoma patients. FASEB Bioadv. 2022; 4:585–601. 10.1096/fba.2022-00017 36089979 PMC9447420

[36] Gao Z, Ling X, Shi C, Wang Y, Lin A. Tumor immune checkpoints and their associated inhibitors. J Zhejiang Univ Sci B. 2022; 23:823–43. 10.1631/jzus.B2200195 36226537 PMC9561405

[37] Liu R, Yang F, Yin JY, Liu YZ, Zhang W, Zhou HH. Influence of Tumor Immune Infiltration on Immune Checkpoint Inhibitor Therapeutic Efficacy: A Computational Retrospective Study. Front Immunol. 2021; 12:685370. 10.3389/fimmu.2021.685370 34220837 PMC8248490

[38] Wei Q, Taskén K. Immunoregulatory signal networks and tumor immune evasion mechanisms: insights into therapeutic targets and agents in clinical development. Biochem J. 2022; 479:2219–60. 10.1042/BCJ20210233 36305711

[39] Wang T, Shen Y, Luyten S, Yang Y, Jiang X. Tissue-resident memory CD8^+^ T cells in cancer immunology and immunotherapy. Pharmacol Res. 2020; 159:104876. 10.1016/j.phrs.2020.104876 32422340

[40] Blake MK, O'Connell P, Aldhamen YA. Fundamentals to therapeutics: Epigenetic modulation of CD8^+^ T Cell exhaustion in the tumor microenvironment. Front Cell Dev Biol. 2023; 10:1082195. 10.3389/fcell.2022.1082195 36684449 PMC9846628

[41] Park J, Hsueh PC, Li Z, Ho PC. Microenvironment-driven metabolic adaptations guiding CD8^+^ T cell anti-tumor immunity. Immunity. 2023; 56:32–42. 10.1016/j.immuni.2022.12.008 36630916

[42] Samstein RM, Lee CH, Shoushtari AN, Hellmann MD, Shen R, Janjigian YY, Barron DA, Zehir A, Jordan EJ, Omuro A, Kaley TJ, Kendall SM, Motzer RJ, et al. Tumor mutational load predicts survival after immunotherapy across multiple cancer types. Nat Genet. 2019; 51:202–6. 10.1038/s41588-018-0312-8 30643254 PMC6365097

[43] Silva SB, Wanderley CWS, Colli LM. Immune Checkpoint Inhibitors in Tumors Harboring Homologous Recombination Deficiency: Challenges in Attaining Efficacy. Front Immunol. 2022; 13:826577. 10.3389/fimmu.2022.826577 35211121 PMC8860897

[44] Garzón-Hernández C, Ramírez-Merino N, Soberon MCM. Molecular Targeted Therapy in Oncology Focusing on DNA Repair Mechanisms. Arch Med Res. 2022; 53:807–17. 10.1016/j.arcmed.2022.11.007 36460545

[45] Ye Z, Shi Y, Lees-Miller SP, Tainer JA. Function and Molecular Mechanism of the DNA Damage Response in Immunity and Cancer Immunotherapy. Front Immunol. 2021; 12:797880. 10.3389/fimmu.2021.797880 34970273 PMC8712645

[46] Park SJ, Ye W, Xiao R, Silvin C, Padget M, Hodge JW, Van Waes C, Schmitt NC. Cisplatin and oxaliplatin induce similar immunogenic changes in preclinical models of head and neck cancer. Oral Oncol. 2019; 95:127–35. 10.1016/j.oraloncology.2019.06.016 31345380 PMC6662630

[47] Blum A, Wang P, Zenklusen JC. SnapShot: TCGA-Analyzed Tumors. Cell. 2018; 173:530. 10.1016/j.cell.2018.03.059 29625059

[48] Vivian J, Rao AA, Nothaft FA, Ketchum C, Armstrong J, Novak A, Pfeil J, Narkizian J, Deran AD, Musselman-Brown A, Schmidt H, Amstutz P, Craft B, et al. Toil enables reproducible, open source, big biomedical data analyses. Nat Biotechnol. 2017; 35:314–6. 10.1038/nbt.3772 28398314 PMC5546205

[49] Yu G, Wang LG, Han Y, He QY. clusterProfiler: an R package for comparing biological themes among gene clusters. OMICS. 2012; 16:284–7. 10.1089/omi.2011.0118 22455463 PMC3339379

[50] Hänzelmann S, Castelo R, Guinney J. GSVA: gene set variation analysis for microarray and RNA-seq data. BMC Bioinformatics. 2013; 14:7. 10.1186/1471-2105-14-7 23323831 PMC3618321

[51] Chen B, Khodadoust MS, Liu CL, Newman AM, Alizadeh AA. Profiling Tumor Infiltrating Immune Cells with CIBERSORT. Methods Mol Biol. 2018; 1711:243–59. 10.1007/978-1-4939-7493-1_12 29344893 PMC5895181

[52] Li T, Fu J, Zeng Z, Cohen D, Li J, Chen Q, Li B, Liu XS. TIMER2.0 for analysis of tumor-infiltrating immune cells. Nucleic Acids Res. 2020; 48:W509–14. 10.1093/nar/gkaa407 32442275 PMC7319575

[53] Luna A, Elloumi F, Varma S, Wang Y, Rajapakse VN, Aladjem MI, Robert J, Sander C, Pommier Y, Reinhold WC. CellMiner Cross-Database (CellMinerCDB) version 1.2: Exploration of patient-derived cancer cell line pharmacogenomics. Nucleic Acids Res. 2021; 49:D1083–93. 10.1093/nar/gkaa968 33196823 PMC7779001

[54] Barretina J, Caponigro G, Stransky N, Venkatesan K, Margolin AA, Kim S, Wilson CJ, Lehár J, Kryukov GV, Sonkin D, Reddy A, Liu M, Murray L, et al. The Cancer Cell Line Encyclopedia enables predictive modelling of anticancer drug sensitivity. Nature. 2012; 483:603–7. 10.1038/nature11003 22460905 PMC3320027

