# Supplementary material for: Integrative pan-cancer analysis reveals the prognostic and immunotherapeutic value of ALKBH7 in HNSC
Source: Aging (Albany NY). 2024 Jun 29;16(19):12781–805. doi: 10.18632/aging.205981 (PMC11501379; doi:10.18632/aging.205981)
Supplement: Supplementary Figures [file aging-16-205981-s001.pdf]

SUPPLEMENTARY FIGURES

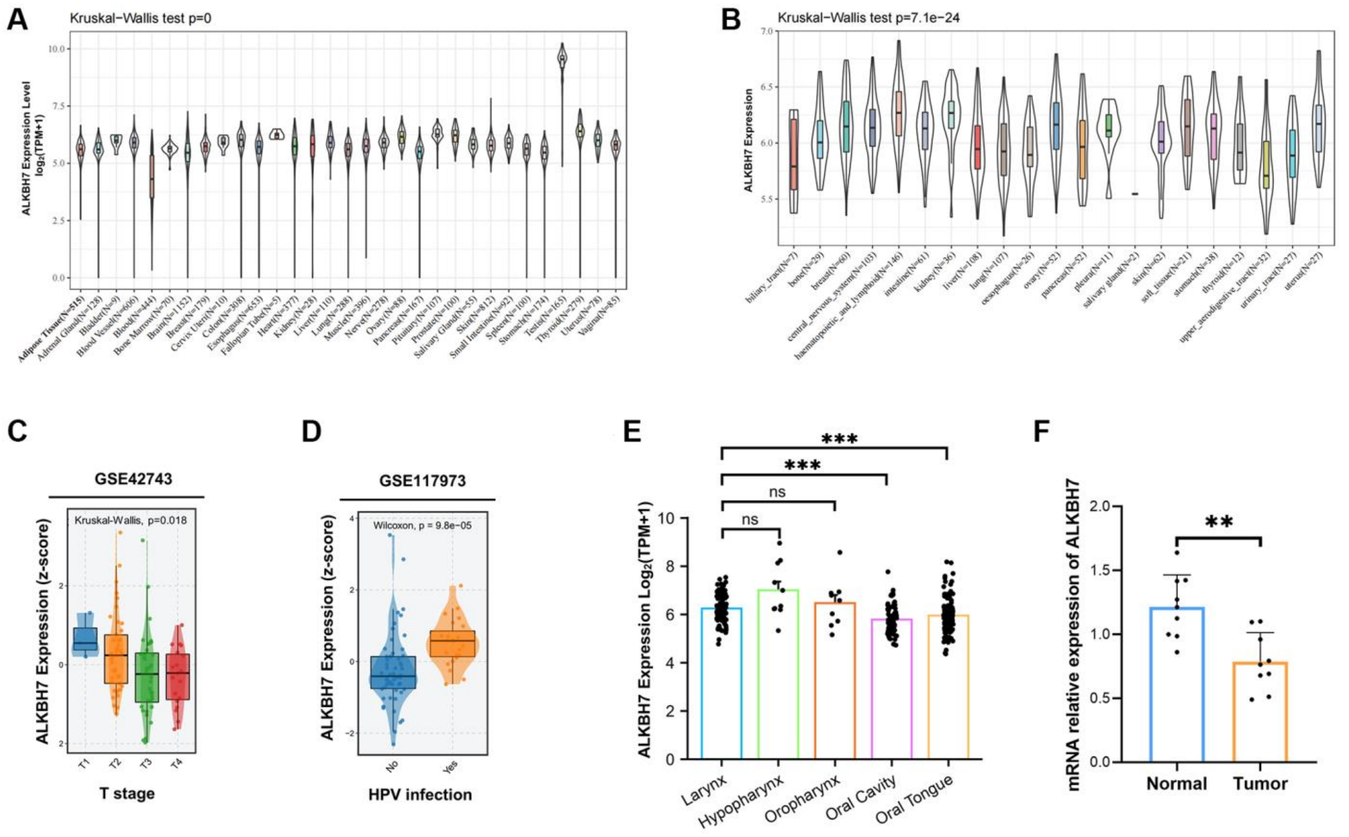

Supplementary Figure 1. Expression levels of ALKBH7 according to various data sources. (**A**) ALKBH7 expression in 31 regular tissues and (**B**) in 21 regular tumor cells. (**C**) The expression level of ALKBH7 in different clinical stages of HNSC. (**D**) The expression level of ALKBH7 in different HPV infection status of HNSC. (**E**) The expression level of ALKBH7 in different anatomic neoplasm subdivision. (**F**) The mRNA relative expression level of ALKBH7 in tumor tissue and adjacent normal tissue from HNSC patients. $^{*}p < 0.05$, $^{**}p < 0.01$.

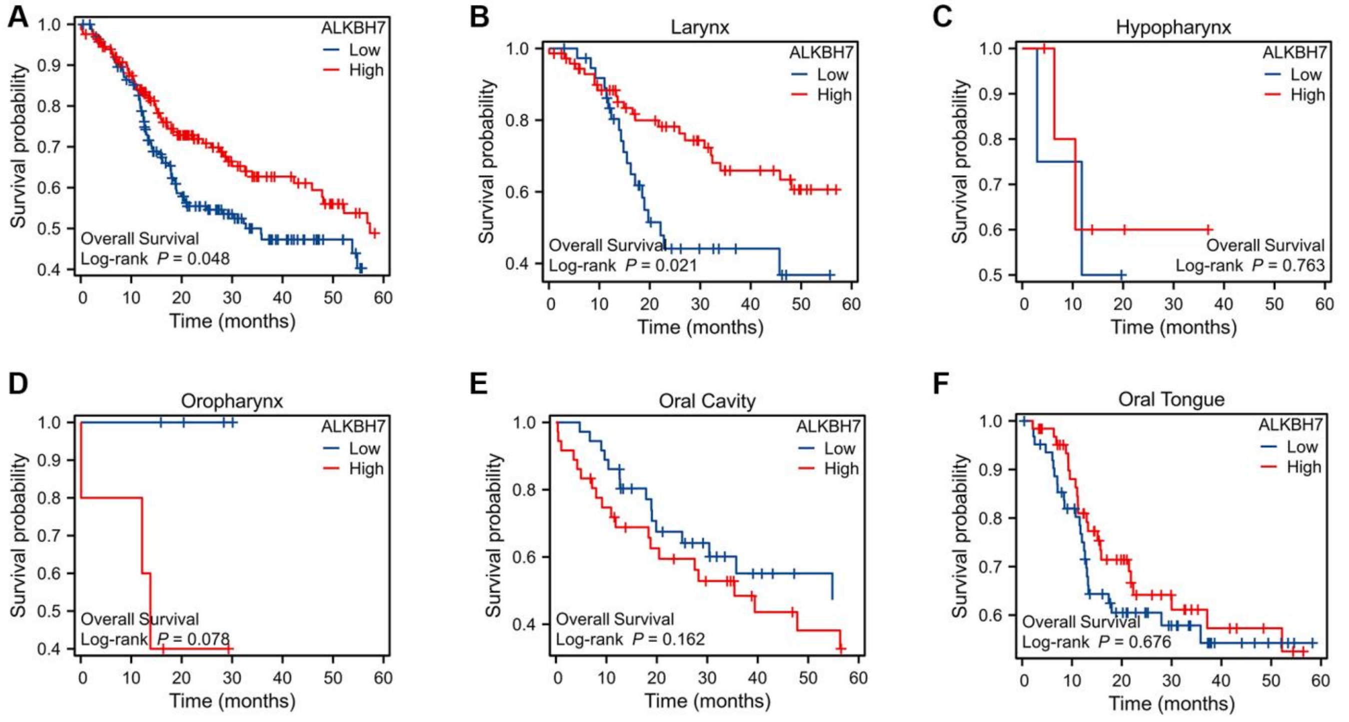

Supplementary Figure 2. Effect of ALKBH7 expression on prognosis in HNSC. (**A**) Correlation between ALKBH7 expression and OS in HNSC patients. (**B**) Correlation between ALKBH7 expression and OS in patients with squamous carcinoma of larynx. (**C**) Correlation between ALKBH7 expression and OS in patients with squamous carcinoma of hypopharynx. (**D**) Correlation between ALKBH7 expression and OS in patients with squamous carcinoma of oropharynx. (**E**) Correlation between ALKBH7 expression and OS in patients with squamous carcinoma of oral cavity. (**F**) Correlation between ALKBH7 expression and OS in patients with squamous carcinoma of oral tongue.

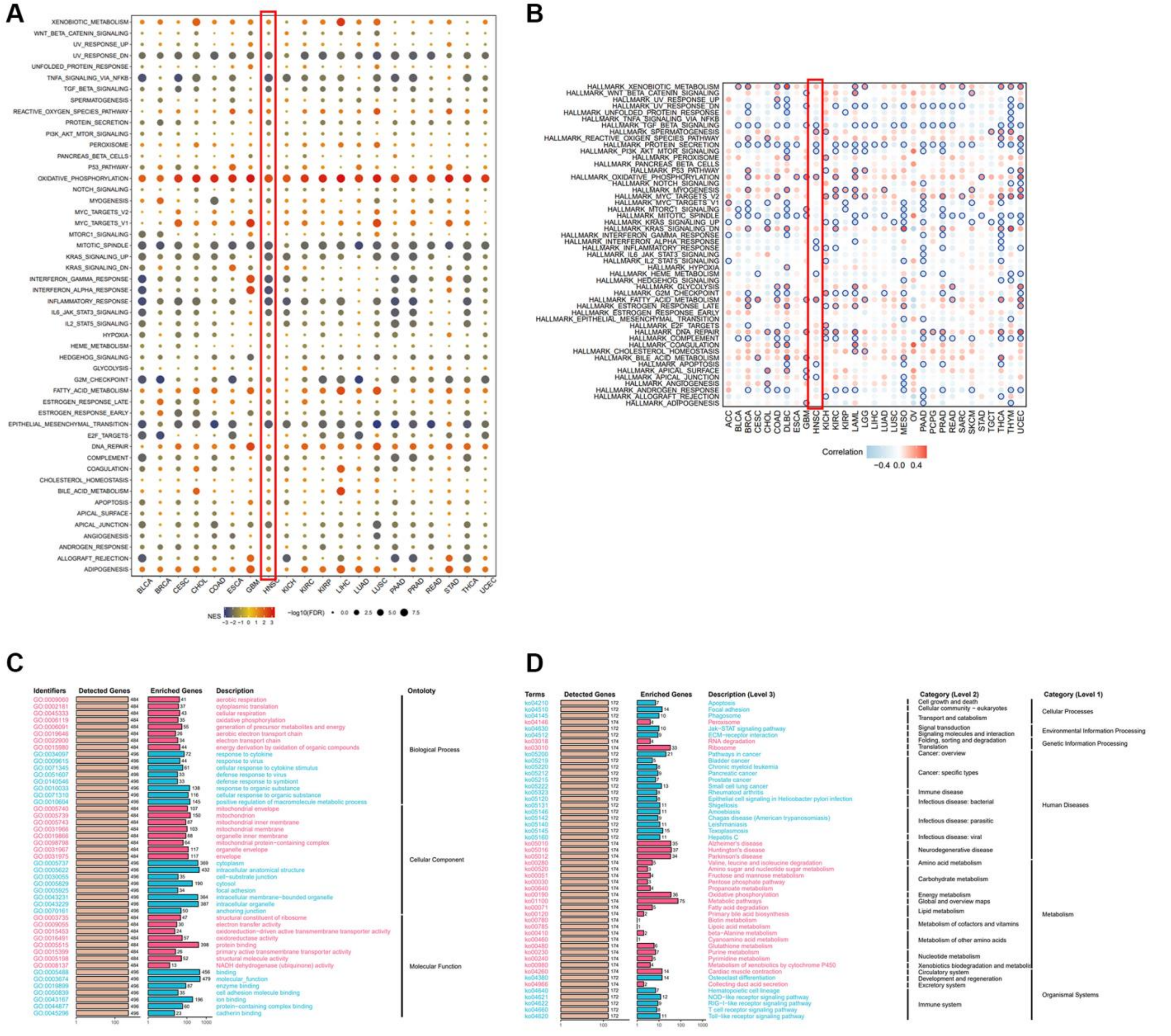

Supplementary Figure 3. Enrichment analysis and visualization of ALKBH7-related partners based on TCGA dataset. (**A**, **B**) GSEA regarding enrichment score group (**A**) and GSVA (**B**) of ALKBH7-related signaling pathways in pan-cancer. (**C**, **D**) Over-representation analysis of ALKBH7-related biological functions using the GO (**C**) and KEGG (**D**) datasets in HNSC. Up-regulated terms appeared in red and down-regulated terms appeared in blue.

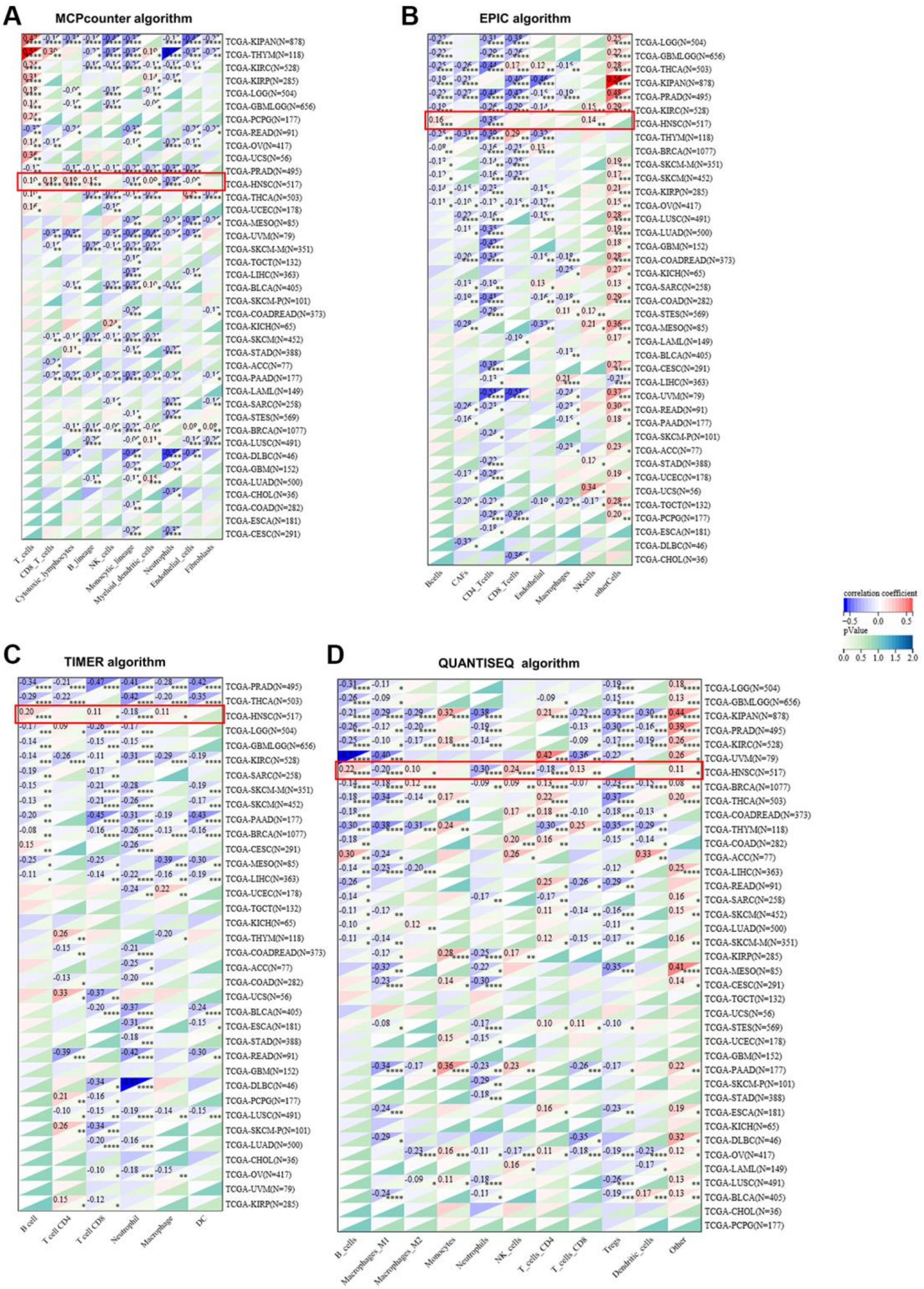

Supplementary Figure 4. Correlationship between ALKBH7 expression and immune cells infiltration based on different data algorithms ((**A**) MCPcounter, (**B**) EPIC, (**C**) TIMER and (**D**) QUANTISEQ). $^{*}p < 0.05$, $^{**}p < 0.01$, $^{***}p < 0.001$, $^{****}p < 0.0001$.

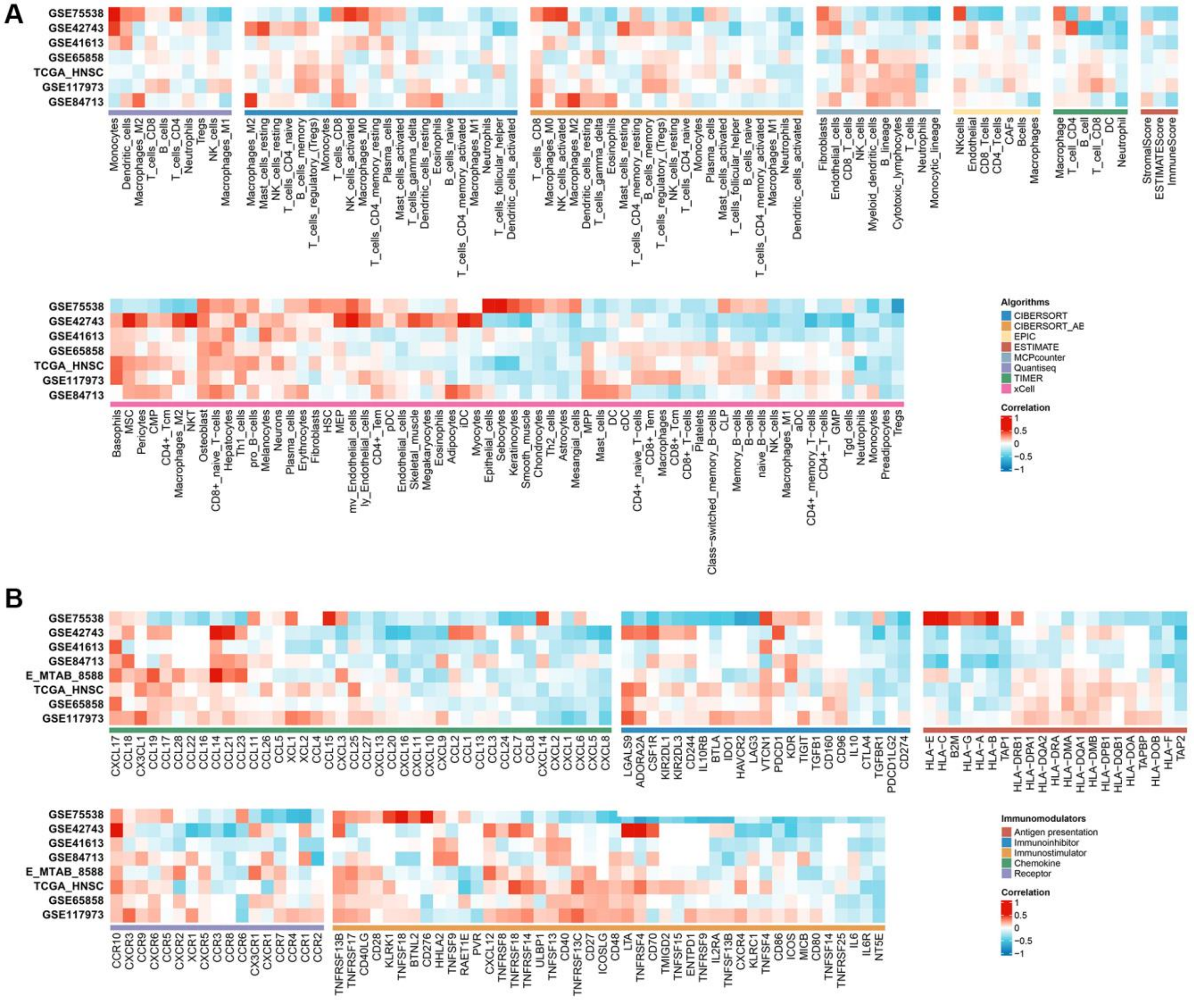

Supplementary Figure 5. Correlations between ALKBH7 expression and immunity in HNSC. (**A**) Correlation of immune cell infiltration among multiple data resource in HNSC. (**B**) Correlation between the expression of ALKBH7 and immunomodulator genes among multiple data resource in HNSC.

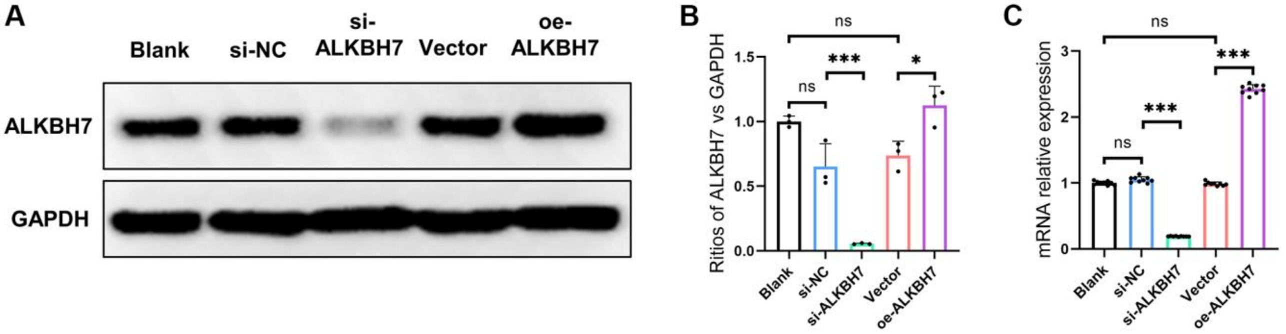

Supplementary Figure 6. Effect of ALKBH7 gene silencing and overexpression in LSC-1. (**A**) Western blotting of ALKBH7 differential expression after gene silencing and overexpression in LSC-1. (**B**) Quantitative ratio of gray value for ALKBH7 protein expressed level assessed by western blotting. (**C**) The mRNA relative expression level of ALKBH7 after gene silencing and overexpression in LSC-1. $^{*}p < 0.05$, $^{**}p < 0.01$, $^{***}p < 0.001$.
