# Supplementary material for: Integrative pan-cancer analysis reveals the prognostic and immunotherapeutic value of ALKBH7 in HNSC
Source: Aging (Albany NY). 2024 Jun 29;16(19):12781–805. doi: 10.18632/aging.205981 (PMC11501379; doi:10.18632/aging.205981)
Supplement: Supplementary Tables [file aging-16-205981-s002.pdf]

SUPPLEMENTARY TABLES

Supplementary Table 1. Clinical characteristics of high and low expression of ALKBH7 in HNSC patients.

Characteristics	Low expression of ALKBH7	High expression of ALKBH7	*P*-value
n	143	133	
Gender, *n* (%)			0.005
Female	51 (18.5%)	27 (9.8%)	
Male	92 (33.3%)	106 (38.4%)	
Age, median (lQR)	61 (53.5, 69)	61 (56,67)	0.732
Smoker, *n* (%)			0.144
No	41 (14.9%)	28 (10.1%)	
Yes	102 (37%)	105 (38%)	
Alcohol history, *n* (%)			0.892
No	43 (15.6%)	39 (14.1%)	
Yes	100 (36.2%)	94 (34.1%)	
Radiation therapy, *n* (%)			0.002
No	60 (21.7%)	32 (11.6%)	
Yes	83 (30.1%)	101 (36.6%)	
Lymphovascular invasion, *n* (%)			0.002
No	106 (38.4%)	75 (27.2%)	
Yes	37 (13.4%)	58 (21%)	
Primary therapy outcome, *n* (%)			0.544
CR	129 (46.7%)	114 (41.3%)	
PR	0 (0%)	1 (0.4%)	
SD	2 (0.7%)	2 (0.7%)	
PD	12 (4.3%)	16 (5.8%)	

Supplementary Table 2. Summary of cancer types.

Cancer abbreviation	Cancer type
ACC	Adrenocortical carcinoma
BLCA	Bladder urothelial carcinoma
BRCA	Breast invasive carcinoma
CESC	Cervical squamous cell carcinoma and endocervical adenocarcinoma
CHOL	Cholangiocarcinoma
COAD	Colon adenocarcinoma
DLBC	Lymphoid neoplasm diffuse large B-celllymphoma
ESCA	Esophageal carcinoma
GBM	Glioblastoma multiforme
HNSC	Head and Neck squamous cell carcinoma
KICH	Kidney chromophobe
KIRC	Kidney renal clear cell carcinoma
KIRP	Kidney renal papillary cell carcinoma
LAML	Acute myeloid leukemia
LGG	Brain lower grade glioma
L1HC	Liver hepatocellular carcinoma
LUAD	Lung adenocarcinoma
LUSC	Lung squamous cell carcinoma

MESO	Mesothelioma
OV	Ovarian serous cystadenocarcinoma
PAAD	Pancreatic adenocarcinoma
PCPG	Pheochromocytoma and Paraganglioma
PRAD	Prostate adenocarcinoma
READ	Rectum adenocarcinoma
SARC	Sarcoma
SKCM	Skin cutaneous melanoma
STAD	Stomach adenocarcinoma
TGCT	Testicular germ cell tumors
THCA	Thyroid carcinoma
THYM	Thymoma
UCEC	Uterine corpus endometrial carcinoma
UCS	Uterine carcinosarcoma
UVM	Uveal melanoma
